# Fine mapping of a *Phytophthora*-resistance locus *RpsGZ* in soybean using genotyping-by-sequencing

**DOI:** 10.1186/s12864-020-6668-z

**Published:** 2020-04-03

**Authors:** Bingzhi Jiang, Yanbo Cheng, Zhandong Cai, Mu Li, Ze Jiang, Ruirui Ma, Yeshan Yuan, Qiuju Xia, Hai Nian

**Affiliations:** 1 The State Key Laboratory for Conservation and Utilization of Subtropical Agro-bioresources, South China Agricultural University, Guangzhou, Guangdong 510642 People’s Republic of China; 2 The Key Laboratory of Plant Molecular Breeding of Guangdong Province, College of Agriculture, South China Agricultural University, Guangzhou, Guangdong 510642 People’s Republic of China; 3 Guangdong Provincial Key Laboratory of Crops Genetics and Improvement, Crops Research Institute, Guangdong Academy of Agricultural Sciences, Guangzhou, 510640 People’s Republic of China; 4 Beijing Genomics Institute (BGI) Education Center, University of Chinese Academy of Sciences, Shenzhen, 518083 People’s Republic of China

**Keywords:** Soybean, *Phytophthora* root rot, Resistance locus, SNP, Fine mapping

## Abstract

**Background:**

*Phytophthora* root rot (PRR) caused by *Phytophthora sojae* (*P. sojae*) is one of the most serious limitations to soybean production worldwide. The identification of resistance gene(s) and their incorporation into elite varieties is an effective approach for breeding to prevent soybean from being harmed by this disease. A valuable mapping population of 228 F_8:11_ recombinant inbred lines (RILs) derived from a cross of the resistant cultivar Guizao1 and the susceptible cultivar BRSMG68 and a high-density genetic linkage map with an average distance of 0.81 centimorgans (cM) between adjacent bin markers in this population were used to map and explore candidate gene(s).

**Results:**

PRR resistance in Guizao1 was found to be controlled by a single Mendelian locus and was finely mapped to a 367.371-kb genomic region on chromosome 3 harbouring 19 genes, including 7 disease resistance (R)-like genes, in the reference Willliams 82 genome. Quantitative real-time PCR assays of possible candidate genes revealed that *Glyma.03 g05300* was likely involved in PRR resistance.

**Conclusions:**

These findings from the fine mapping of a novel *Rps* locus will serve as a basis for the cloning and transfer of resistance genes in soybean and the breeding of *P. sojae-*resistant soybean cultivars through marker-assisted selection.

## Background

*Phytophthora* root rot (PRR) caused by *Phytophthora sojae* is one of the most important soil-borne diseases in many soybean-producing regions of the world and causes significant soybean production losses [1].

Soybean resistance to *P. sojae* is mainly controlled by two mechanisms, involving complete or partial resistance genes [2, 3]. The former type of resistance is related to a single dominant resistance gene [4–19], with *P. sojae* interacting with *Rps* genes in a gene-for-gene system preventing disease development in plants [20], while the latter involves multiple genes and limits damage to the plant [3, 21].

To our knowledge, more than 33 *Rps* genes/alleles on 9 different soybean chromosomes have been identified and mapped, among which *Rps1* (including five alleles, *Rps1a*, *Rps1b*, *Rps1c*, *Rps1d* and *Rps1 k*), *Rps7*, *Rps9*, *RpsYu25*, *RpsYD29*, *RpsWY, RpsUN1*, *RpsHN*, *RpsHC18*, *RpsQ*, *RpsX* and an unnamed *Rps* gene in Wascshiroge and E00003 soybean were mapped to chromosome 3 [7, 9, 10, 13, 14, 22–31]. *Rps3* (including three alleles, *Rps3a*, *Rps3b* and *Rps3c*) and *RpsSN10* were mapped to chromosome 13, which is linked with *Rps8* [6, 22, 32, 33]. *Rps2* and *RpsUN2* were found on chromosome 16 [22, 28]. Additionally, *Rps4*, *Rps5*, *Rps6, Rps12* and *RpsJS* are located on chromosome 18 [16, 19, 22, 34, 35], and *RpsYB30*, *RpsZS18*, *RpsSu*, *Rps10* and *Rps11* are located on chromosomes 19, 2, 10, 17 and 7, respectively [8, 11, 18, 36, 37]. Among the identified *Rps* genes on chromosome 3, *Rps1 k* was mapped to a 125-kb region and cloned and was found to show an NBS-LRR structure that is typical of a resistance protein [24, 38]. *RpsYD29* was mapped to a 204.8-kb region, and two nucleotide-binding site and leucine-rich repeat (NBS-LRR)-type genes, *Glyma03g04030.1* and *Glyma03g04080.1,* were identified [27]. Moreover, *RpsQ* was finely mapped to a 118-kb region [13].

Recently, with the progress of massively parallel DNA sequencing platforms, whole-genome sequencing (WGS) has become the primary strategy for next-generation sequencing (NGS) for SNP discovery and genotyping in large populations. These methods include resequencing, genotyping by-sequencing (GBS) [39], specific length amplified fragment sequencing (SLAF-seq) [40], restriction site-associated DNA tag sequencing (RAD-seq) [41], and 2b-RAD [42]. NGS technologies have been widely utilized in soybean, wheat, sunflower and other crops to develop SNP markers and map genes/QTLs [11, 43–47]. The dominant soybean *Phytophthora* root rot resistance gene *RpsWY* was mapped using a high-density soybean genetic map comprising 3469 recombination bin markers using RAD-seq technology in 196 F_7:8_ RILs [31].

In this study, we found that the cultivar Guizao1 presented broad-spectrum resistance and may carry *Rps* genes or alleles. The objectives of our project were to characterize the inheritance of the *Rps* gene(s) and finely map the candidate gene(s) of the resistant cv. Guizao1 using a high-density genetic linkage map comprising 3748 recombination bin markers using RAD-seq technology in 228 F_8_ RILs derived from a cross of Guizao1 × BRSMG68.

## Results

### Phenotype reaction of the parents to *P. sojae* isolates

To investigate the phenotypes of Guizao1 and BRSMG68, six isolates of *P. sojae* were used to test the reactions of the genetically different soybean varieties (Table 1). The inoculation results showed that BRSMG68 showed the same SSSSSS reaction as Williams, indicating that BRSMG68 did not contain known disease resistance genes. Guizao1 (RSRSSS), Chapman (RRRRSR), L85–3059 (RSRSSR) and Harosoy (RSSSSS) were PRR resistant to the *P. sojae* PNJ4 strain, while other varieties were PRR susceptible to the PNJ4 strain (Table 1). Furthermore, Guizao1 also PRR resistant to the PNJ1 strain but PRR susceptible to the Pm28, PNJ3, Pm14, and P6497 strains, which was different from what was observed for Chapman, L85–3059 and Harosoy. The inoculation results suggest that Guizao1 may contain a novel *Rps* gene or resistance locus.

**Table 1:** Differential reactions of soybean hosts and cultivars to strains of *P. sojae*

Cultivar	*Rps*	*Phytophthora sojae* strains					
		PNJ4	Pm28	PNJ1	PNJ3	Pm14	P6497
Guizao1		R	S	R	S	S	S
BRSMG68		S	S	S	S	S	S
Harlon	*1a*	S	S	R	S	S	R
Harosoy13XX	*1b*	S	S	R	S	S	S
Williams79	*1c*	S	S	R	S	S	R
PI103091	*1d*	S	S	S	S	S	R
Williams82	*1 k*	S	S	R	S	S	R
L76–988	*2*	S	S	S	S	S	S
Chapman	*3a*	R	R	R	R	S	R
PRX146–36	*3b*	S	S	S	S	R	R
PRX145–48	*3c*	S	S	S	S	S	S
L85–2352	*4*	S	R	S	R	S	R
L85–3059	*5*	R	S	R	S	S	R
Harosoy62XX	*6*	S	S	S	R	S	R
Harosoy	*7*	R	S	S	S	S	S
Williams	*rps*	S	S	S	S	S	S

### Genetic analysis of resistance to *P. sojae* PNJ4

Among the 228 F_8:11_ RILs obtained from the cross of Guizao1 × BRSMG68, 113 RILs were homozygous resistant, and 115 RILs were homozygous susceptible, with the segregation ratio fitting with the Mendelian genotypic ratio of 1R:1S (X^2^ = 0.004, *P* = 0.95, Table 2). These results indicated that PRR resistance in Guizao1 was controlled by a single locus, which we temporarily designated as *RpsGZ*.

**Table 2:** Segregation analysis of resistance to *P. sojae* PNJ4 in F_8:11 (_Guizao1 × BRSMG68)

Cross or Parent^a^	Total no. of plants/lines		Expected ratio and goodness of fit		
	Resistance	Susceptibility	Expected ratio	X^2^	P
BRSMG68	0	180			
Guizao1	180	0			
F_8:11(_Guizao1 × BRSMG68)	113	115	1:1	0.004	0.95

(^a^) BRSMG68 was PRR-susceptible cultivars to PNJ4 strain, and Guizao1 was PRR-resistant to PNJ4 strain

### Fine mapping of *RpsGZ* by high-throughput genome-wide resequencing

**Fig. 1:**
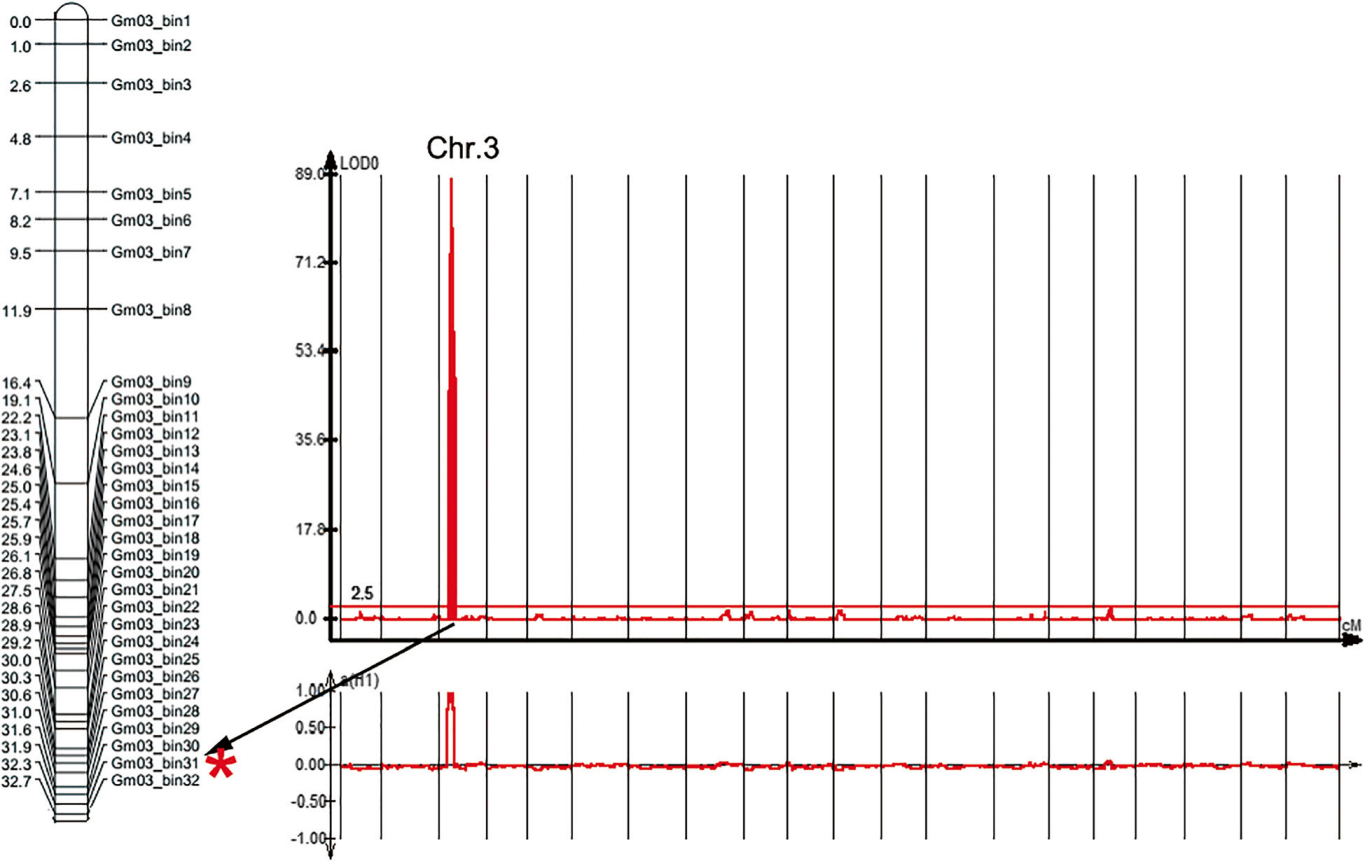
Results of *RpsGZ* locus analysis using the CIM method in the F_8:11_ RILs. The LOD value distribution in the whole genome of the RIL population from a cross of Guizao1 × BRSMG68. *RpsGZ* was amplified at the site of bin31 on chromosome 3, which explained 81.8% of the phenotypic variance

**Fig. 2:**
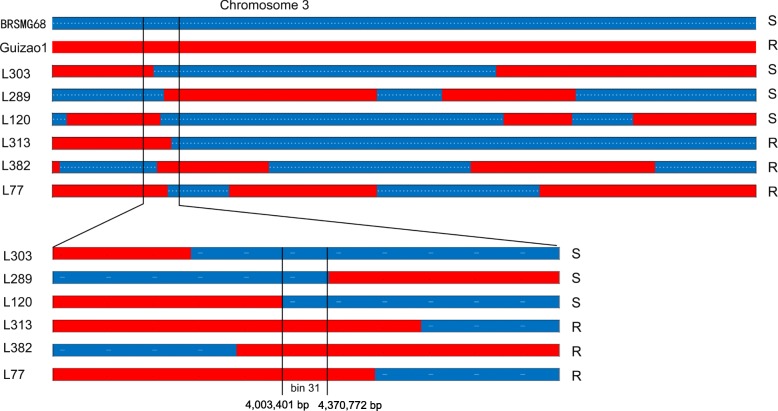
Fine mapping of the *RpsGZ* locus. Recombinant inbred lines showing recombination near the *RpsGZ* locus are shown with blue and red bars representing homozygous genotypes from BRSMG68 and Guizao1, respectively. Line 120, 289 and 303 were PRR-susceptible plants (S). Line 77, 313 and 382 were PRR-resistant plants (R)

Based on the high-density map constructed with bins as markers and the use of CIM with WinQTLCart for PRR resistance locus localization, only one PRR resistance locus was detected on chromosome 3 in Guizao1 (Fig. 1), with a log-likelihood (LOD) value of 88.28, which explained 81.75% of the phenotypic variance. In the high-density linkage map (Additional file: Fig. S1), *RpsGZ* was placed in bin31 according to the results for six recombinant monoclonal lines (Fig. 2). This placed *RpsGZ* in a region between 4,003,401 and 4,370,772 bp in GlymaWm82.a2.v1, covering appropriately 367,371 bp. A BLAST search showed 19 annotated genes based on this assembly (Table 3; http://www.soybase.org). The putative functions of these predicted genes were annotated via BLAST searches against the TAIR protein datasets and the Phytozome Genomics Resource (https://phytozome.jgi.doe.gov/pz/portal.html), and five genes (*Glyma.03G034400*, *Glyma.03G034500*, *Glyma.03G034800*, *Glyma.03G034900* and *Glyma.03G035300*) were found to contain nucleotide-binding site (NBS)-leucine-rich repeat (LRR) domains, which are important domains of plant disease resistance genes. *Glyma.03G035900* is a membrane attack complex/perforin (MACPF) domain-encoding gene, and the MACPF proteins play a role in immunity (http://pfam.xfam.org). *Glyma.03G036000* encodes a serine/threonine protein kinase that plays an important role in signalling and plant defence activities. Therefore, these seven R-like genes were most likely the candidate genes of *RpsGZ*.

**Table 3:** Annotations of the candidate genes in the *RpsGZ* region on chromosome 3

No	Gene name^a^	Annotation	Ortholog^b^
1	*Glyma.03G034400*	Disease resistance protein (NBS-LRR class), putative^c^	AT3G14470.1
2	*Glyma.03G034500*	Disease resistance protein (NBS-LRR class), putative	AT3G14470.1
3	*Glyma.03G034600*	No items to show	AT1G62130.1
4	*Glyma.03G034700*	No items to show	AT2G01050.1
5	*Glyma.03G034800*	Disease resistance protein (NBS-LRR class), putative	AT3G14470.1
6	*Glyma.03G034900*	Disease resistance protein (NBS-LRR class), putative	AT3G14470.1
7	*Glyma.03G035000*	Domain of unknown function DUF223	AT2G05642.1
8	*Glyma.03G035100*	PIF1-like helicase	AT3G51690.1
9	*Glyma.03G035200*	CW-type Zinc Finger; B3 DNA binding domain	AT4G32010.1
10	*Glyma.03G035300*	Disease resistance protein (NBS-LRR class), putative. Protein tyrosine kinase	AT3G08760.1
11	*Glyma.03G035400*	PPR repeat	AT3G42630.1
12	*Glyma.03G035500*	Plant mobile domain	AT2G04865.1
13	*Glyma.03G035600*	Protease inhibitor/seed storage/LTP family	AT3G08770.1
14	*Glyma.03G035700*	No items to show	AT5G59310.1
15	*Glyma.03G035800*	Pollen allergen; Rare lipoprotein A (RlpA)-like double-psi beta-barrel	AT5G05290.1
16	*Glyma.03G035900*	Membrane attack complex/Perforin domain	AT1G29690.1
17	*Glyma.03G036000*	Protein tyrosine kinase; Serine-threonine protein kinase	AT5G01850.1
18	*Glyma.03G036100*	No items to show	
19	*Glyma.03G036200*	Multidrug resistance protein	AT2G38510.1

**(**^**a**^**)** Glyma ID from the Williams 82 soybean reference genome Wm82.a2.v1 (http://soybase.org)

**(**^**b**^**)** Accession number of Arabidopsis orthologs were obtained from the Arabidopsis Information Resource (TAIR10, http://www.arabidopsis.org/)

**(**^**c**^**)** NBS-LRR: Nucleotide-binding site (NBS) -leucine-rich repeat (LRR) domains

### Gene ontology (GO) enrichment analysis of the candidate genes

The AgriGO toolkit was used to perform gene ontology (GO) analysis [48, 49]. Among the 19 genes in the region close to *RpsGZ* detected in this study, 9 genes were found to show at least one GO annotation (Additional file: Fig. S2, Table S1 and Table S2). These genes were predicted to be involved in biological processes and molecular functions including protein kinase activity, protein amino acid phosphorylation, ribonucleotide binding, cellular processes, ADP binding, and nucleoside binding. In the molecular function category, the GO terms “adenyl nucleotide binding” “purine ribonucleotide binding” and “adenyl ribonucleotide binding” were significantly enriched (Fig. S2). Among the GO terms, both *Glyma.03G035300* and *Glyma.03G036000* were associated with the term “GO:0006468 protein amino acid phosphorylation”. Protein phosphorylation is a ubiquitous mechanism for modulating protein function [50] and plays a role in defence mechanisms against disease.

### Expression profiling for the identification of resistance genes

**Fig. 3:**
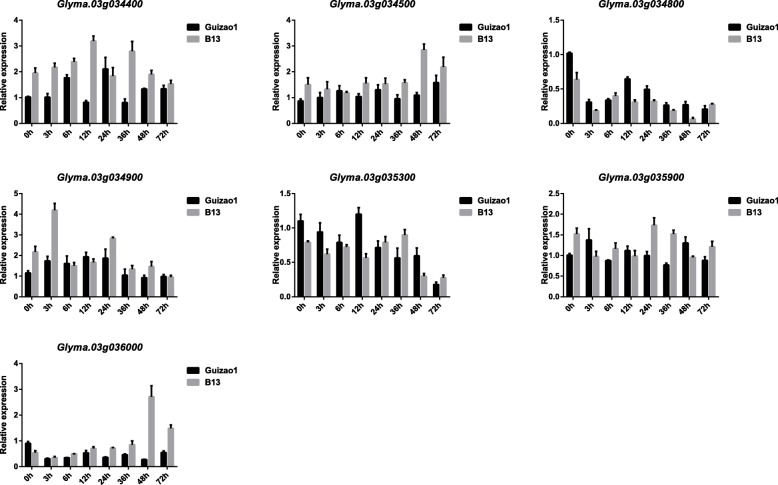
Relative expression levels of the candidate genes of the *RpsGZ* locus. Y-axes indicate the ratios of the relative fold differences in expression levels between samples infected with *P. sojae* PNJ4. The primary leaf samples were harvested at 0, 3, 12, 24, 36, 48, and 72 h post-inoculation. The transcript levels of the candidate genes of the *RpsGZ* locus were assessed by qRT-PCR using the 2^–∆∆Ct^ method with the actin gene as an internal control

To confirm which genes were induced under infection with *P. sojae*, the expression patterns of 7 R-like genes were examined in Guizao1 and BRSMG68 using qRT-PCR analysis (Fig. 3). The expression levels of four genes (i.e., *Glyma.03G034400*, *Glyma.03G034500*, *Glyma.03G034900* and *Glyma.03G035900*) were upregulated at most time points after infection in Guizao1 and BRSMG68. However, the other genes (*Glyma.03 g034800*, *Glyma.03 g035300* and *Glyma.03 g036000*) were downregulated at 3, 6, 24 and 36 h after treatment in Guizao1 and BRSMG68.

The expression of *Glyma.03G034400*, *Glyma.03G034500*, *Glyma.03G034900* and *Glyma.03 g036000* in the susceptible cv. BRSMG68 was higher than in the resistant cv. Guizao1 at most time points after infection. The expression of *Glyma.03G035300* in Guizao1 was higher than that in BRSMG68 at 3, 6, and 12 h after treatment, reaching a maximum expression increase of approximately 2.1-fold at 12 h after treatment, followed by a decrease from 24 to 72 h after treatment. A similar expression pattern was observed for the *Glyma.03G034800* gene, with a relatively low expression level. These results showed that the *Glyma.03G035300* gene may be involved in disease-defence mechanisms.

## Discussion

Soybean is one of the most important crops in the world. There are a large number of soybean accessions in China, among which many PRR-resistant cultivars/lines were identified in a previous study [10, 13, 14, 51–55]. In the present study, the Guizao1 cultivar was PRR resistant to *P. sojae* PNJ4 and PNJ1, thus differing from the other soybean cultivars tested (Table 1). Genetic analyses indicated that resistance to *P. sojae* PNJ4 in Guizao1 was controlled by a single locus.

To more finely map the PRR resistance locus, *RpsGZ* was mapped in an RIL population based on genotyping through resequencing, resulting in the integration of 54,002 SNPs into 3748 recombination bin units. These markers were then employed to construct a high-density bin linkage map with an average distance of 0.81 cM between adjacent markers [56]. The map exhibited well-distributed linkage distances and a higher resolution than the conventional map, and gene/QTL mapping was thus more accurate and reliable. The position of *RpsGZ* was refined through fine mapping to a 367,371 bp interval between 4,003,401 and 4,370,772 bp on chromosome 3, which was the region rich in *Rps* genes.

Previous studies have identified 17 known *Rps* genes (alleles) and mapped them to chromosome 3 before *RpsGZ*, including five alleles of *Rps1* (*Rps1a, 1b, 1c, 1d, 1 k*) [23, 24, 38, 57, 58], *Rps7* [23], *Rps9* [29], *RpsYu25* [25], an *Rps* gene in Waseshiroge [26], *RpsYD29* [27], an *Rps* gene in E00003 soybean within the *Rps1 k* interval [30], *RpsHC1*8 [10], *RpsQ* [13], *RpsHN* [14], *RpsX* [9], *RpsWY* [31], and *RpsUN1* [28]. Nevertheless, the positional relationships of these *Rps* genes had not been confirmed, and some of the mapping intervals for these *Rps* genes overlapped. Therefore, whether these genes were allelic or located at a new locus needed to be confirmed.

In the present study, *RpsGZ* was found to be a distinct gene from the *Rps1* alleles because five varieties carrying *Rps1* (*1a*, *1b*, *1c*, *1d* and *1 k*) were PRR susceptible to *P. sojae* PNJ4, although the candidate region of *RpsGZ* partly overlapped with the region of *Rps1*. The Wayao cultivar (*RpsWY*) was susceptible to *P. sojae* PNJ4, Guizao1 was resistant to *P. sojae* PNJ4 [31], and these two mapping parents exhibited different resistance reactions, suggesting that *RpsGZ* may be different from *RpsWY*. Compared with the nucleotide positions of the *Rps* genes mapped to chromosome 3 (Table 4) according to the Glyma 2.0 soybean gene annotation database (http://soybase.org/), the positional information for *RpsGZ* suggested that *RpsGZ* was distinct from 9 known *Rps* genes, including *Rps1a*, *Rps1b*, *Rps1c, Rps1d*, *Rps9*, *RpsQ*, *RpsX*, *RpsYu25* and *RpsHC18.*

**Table 4:** The location of *Rps* genes on chromosome 3

No	*Rps* gene	Molecular marker interval		Physical posistion (bp)		
1	***RpsGZ***			4,003,401	–	4,370,772^a^
2	*RpsX*			2,910,913	–	3,153,254^a^
3	*Rps9*	Satt631	Satt152	2,943,883	–	3,366,655^a^
4	*RpsQ*	BARCSOYSSR_03_0165	InDel281	2,968,566	–	3,087,579^a^
5	*Rps1a*	Satt159	Satt009	3,197,845	–	3,932,116^a^
6	*RpsYu25*	Satt152	Sat_186	3,366,405	–	3,488,905^a^
7	*Rps1d*	Satt152	Sat_186	3,366,405	–	3,488,905^a^
8	*Rps1*	Sat_186	Satt530	3,488,616	–	5,669,877^a^
9	*RpsYD29*	SattWM82–50	Satt1 k4b	3,857,715	–	4,062,474^b^
10	*Rps7*	Satt009	Satt125	3,931,955	–	18,415,710^a^
11	*Rps* gene in Waseshiroge	Satt009	T003044871	3,910,260	–	4,486,048^b^
12	*RpsUN1*	BARCSOYSSR_03_0233	BARCSOYSSR_03_0246	4,020,587	–	4,171,402^a^
13	*RpsHN*	SSRSOYN-25	SSRSOYN-44	4,227,863	–	4,506,526^b^
14	*Rps1 k*			4,457,810	–	4,641,921^b^
15	*RpsWY*			4,466,230	–	4,502,773^b^
16	*Rps* gene in E00003			4,475,877	–	4,563,799^b^
17	*RpsHC18*	BARCSOYSSR_03_0265	BARCSOYSSR_03_0272	4,446,594	–	4,611,282^a^
18	*Rps1b*	Satt530	Satt584	5,669,877	–	9,228,144^a^
19	*Rps1c*	Satt530	Satt584	5,669,877	–	9,228,144^a^

**a**:the nucleotide positions of the markers were determined through a BLAST search in the Glyma 2.0 soybean gene annotation database (http://soybase.org/); **b**:the nucleotide positions of the markers were determined according to the Glyma 1.0 soybean gene annotation database (http://soybase.or

In addition, *Rps7* was mapped to a 14,483,755 bp genomic region (3,931,955–18,415,710 bp) flanked by the SSR markers Satt009 and Satt125 [23]. *RpsUN1* was localized to the region between 4,020,587 and 4,171,402 bp, flanked by two SSR markers, BARCSOYSSR_03_0233 and BARCSOYSSR_03_0246, based on the Glyma 2.0 soybean gene annotation database of the Williams 82 genome sequence [28]. Among the regions of four other known *Rps* genes according to the Glyma1.0 annotations, the Waseshiroge *Rps* gene was located between Satt009 and T003044871 and may reside in the nucleotide region between 3,910,260 and 4,486,048 bp of the Williams 82 genome [26]. The *Rps* gene in cv. E00003 was positioned within the interval of 4,475,877 to 4,563,799 bp [30]. *RpsHN* was mapped to a 278.7 kb genomic region flanked by the SSR markers SSRSOYN-25 and SSRSOYN-44 and may reside at nucleotide position 4,227,863 and 4,506,526 bp [14]. *RpsYD29* was flanked by the markers SattWM82–50 and Satt1 k4b, which were located at nucleotide positions 3,857,715 and 4,062,474 bp [27]. *RpsGZ* was also located in a region between 4,022,530 and 4,483,231 bp in GlymaWm82.a1.v1. Therefore, *RpsGZ* and the *Rps7*, *RpsHN, RpsUN1, RpsYD29*, and *Rps* genes from Waseshiroge and E00003 may be tightly linked genes, different alleles of the same gene, or identical alleles of the same gene. However, further confirmation is needed. Moreover, if the sources of resistance mentioned above carry different resistance genes, a pyramiding effect of different resistance genes may increase the resistance of soybean cultivars to *P. sojae*.

The NBS-LRR genes are the extremely large family of plant disease resistance genes [59], and the local tandem duplication of NBS genes has created many homogenous clustered loci in each legume genome studied to date [60]. Meziadi et al. suggested that the NBS-LRR proteins are encoded by one of the largest and most variable multigene families and are often organized into complex clusters of tightly linked genes in plants [61]. In soybean, 319 putative NBS-LRR genes and 175 disease resistance QTLs have been found, among which 36 NBS-LRR genes are clustered on chromosome 3, and most of the NBS-LRR genes are located at the front end of chromosome 3 [62]. The 17 identified *Rps* genes were all mapped to regions between 2,943,883 and 9,228,144 bp on chromosome 3. In addition, some genes or QTLs for resistance to abiotic or biotic stresses in soybean have been mapped near the region of *RpsGZ* on chromosome 3. For instance, the QTL *Raso1* for major foxglove aphid resistance was mapped to a 63-kb interval containing an NBS-LRR-type R-like gene and two other genes in the Williams 82 sequence assembly [63]. A minor foxglove aphid resistance QTL in PI 366121 [64], two soybean sudden death syndrome resistance QTLs, *di1* [65, 66] (also known as *qRfs6* [67]) and *SDS14–1* [68], and the major QTLs or dominant loci underlying salt tolerance in the soybean cultivars Tiefeng8 and Jidou12 [69, 70] might be clustered in the region as *Rps* resistance genes. Among the 19 genes in the region close to *RpsGZ* detected in this study, five gene candidates were NB-ARC domain and leucine-rich repeat-containing (NBS-LRR) genes, which are a typical type of so-called R-genes. NBS-LRR-type genes have been implicated in the resistance of *Rps1 k* [38]. qRT-PCR analysis showed differential expression patterns of the NBS-LRR-type gene *Glyma.03 g05300* between Guizao1 and BRSMG68, and this gene may be involved in defence mechanisms against disease.

## Conclusions

We identified and finely mapped a novel *Rps* locus (*RpsGZ*) that can confer resistance to *P. sojae PNJ4* and *PNJ1* on chromosome 3, which could be used for the breeding of *Phytophthora*-resistant cultivars. The R-like gene *Glyma.03 g05300* may be involved in disease-defence mechanisms. This study provides information regarding the genetic location of the *Rps* resistance locus, which is useful for breeders to apply marker-assisted selection (MAS) in soybean breeding programmes to achieve resistance to *P. sojae*.

## Methods

### Plant materials

The mapping populations of 228 F_8:11_ recombinant inbred lines (RILs) derived from a Guizao1 (P_1_, PRR resistance) × BRSMG68 (P_2_, PRR susceptible) cross were developed via the single-seed descent method [71]. The soybean cv. Guizao1 was developed in Guangxi, China. BRSMG68 was introduced from Brazil. Both cv. Guizao1 and BRSMG68 were obtained from the Guangdong Subcenter of the National Center for Soybean Improvement, South China Agricultural University.

To determine which *Rps* gene or *Rps* gene combination was present in Guizao1, a differential set of 13 cultivars/genotypes was used. Each cultivar/genotype carried a single known *Rps* gene: Harlon (*Rps1a*), Harosoy13XX (*Rps1b*), Williams79 (*Rps1c*), PI103091 (*Rps1d*), Williams82 (*Rps1 k*), L76–988 (*Rps2*), L83–570 (*Rps3a*), PRX146–36 (*Rps3b*), PRX145–48 (*Rps3c*), L85–2352 (*Rps4*), L85–3059 (*Rps5*), Harosoy62XX (*Rps6*) and Harosoy (*Rps7*). The variety Williams (no known *Rps* gene) was used as a susceptible variety to verify successful inoculation. All the different hosts used for PRR identification were kindly provided by the National Center for Soybean Improvement, Nanjing Agricultural University.

### *P. sojae* isolates

Six *P. sojae* isolates (PNJ4, Pm14, Pm28, PNJ1, PNJ3, P6497), which were provided by Prof. Yuanchao Wang and Han Xing at Nanjing Agricultural University were preserved on V8 juice agar medium (10% V8 vegetable juice, 0.02% CaCO_3_ and 1.0% Bacto-agar) [12, 14]. These *P. sojae* isolates were used in the phenotype test of disease resistance to PRR among Guizao1 and BRSMG68 and 13 different cultivars/genotypes. The *P. sojae* PNJ4 strain (virulence formula is *1a, 1b, 1c, 1d, 1 k, 2, 3b, 3c, 4, 6*) was used to evaluate the RIL population of Guizao1 × BRSMG68.

### Evaluation of genetic materials for *Phytophthora* resistance

The method of injured hypocotyl inoculation with slight modification was utilized for disease evaluation in this experiment [12, 25]. For inoculation with *P. sojae*, 15 plants of each host used for PRR identification and each line of the RIL population and the parents were planted in 11.8-cm-diameter plastic pots filled with vermiculite and kept in an illumination incubator (26/22 °C day/night temperature, 75% relative humidity, 12 h light/12 h dark photoperiod and average light intensity of ~ 10,000 Lx).

Seven days after sowing, the seedlings were inoculated. A thinner uniform wound was cut in the soybean cotyledon under a section of 1–2 cm by using a single-sided blade sterilized with the outer flame of an alcohol lamp. The active edges of the colonies of the *P. sojae* isolates cultured in the incubator at 25 °C for 5 days were cut into approximately 3 mm^2^ blocks and then embedded in the wound with the mycelium surface inward. After inoculation, the seedlings were placed on a culture shelf surrounded by plastic film; the moisture content was maintained at 90% relative humidity and the temperature at 25 °C for 24 h, and the plants were sprayed with sterile water, then transferred to the illumination incubator (26/22 °C day/night temperature, 75% relative humidity, 12 h light/12 h dark photoperiod and average light intensity of ~ 10,000 Lx). The evaluation test was set up with three biological experiments. To test the phenotypes of the RIL population inoculated with the PNJ4 isolate, Williams (*rps* gene) was used as the susceptible cultivar to indicate successful inoculation. All the experiments were performed in 2017 and 2018 at South China Agricultural University.

The reactions of all materials were recorded as the percentage of dead seedlings at 5 days after inoculation. Cultivars for PRR identification were considered resistant if all the seedlings were alive with no expanded lesions. Cultivars were considered susceptible if all the seedlings were dead. The RIL families with 70–100% dead seedlings were considered to be homozygous susceptible (S), while the RIL families with 0–30% dead seedlings were considered to be resistant (R), and the RIL families with 31–69% dead seedlings were considered to be heterozygous resistant (Rs) [12].

### Data analysis and candidate gene prediction

A goodness-of-fit to the Mendelian segregation ratio was calculated via Chi-square (X^2^) analysis to examine the segregation patterns of the phenotypes.

The linkage map used in this study was constructed previously by the Guangdong Subcenter of National Center for Soybean Improvement, South China Agricultural University. This map included 3748 bins and was 3031.9 cM in length, with an average distance of 0.81 cM between adjacent markers on 20 chromosomes [56]. Composite interval mapping (CIM) was used to detect gene/QTL-associated PRR resistance using phenotypic data via in WinQTLCart 2.5 software (http://statgen.ncsu.edu/qtlcart/WQTLCart.htm). The LOD thresholds for gene/QTL significance were determined by a test (1000 replications) with a genome-wide scope at the 5% level of significance. Gene mapping results were compared to SoyBase, and the predicted genes in the target regions were analysed according to the annotation of the soybean reference genome (Wm82.a2.v1) in Soybase (http://www.soybase.org/). The functional predictions of genes were manually confirmed by using the BLAST function in the NCBI (http://www.ebi.ac.uk/Tools/sss/ncbiblast/) and TAIR protein datasets (http://www.arabidopsis.org/).

### Expression analysis of candidate genes

Guizao1 and BRSMG68 seedlings were cultivated for 7 days and inoculated with *P. sojae* PNJ4, then transferred to the illumination incubator at 25 °C, 75% relative humidity and a 12 h light/12 h dark photoperiod. Total RNA was isolated from the primary leaves at 0, 3, 12, 24, 36, 48, and 72 h postinoculation. Total RNA was extracted from the plants using TRIzol reagent (Invitrogen, USA) according to the manufacturer’s instructions, and 1 μg of DNase-treated RNA was subjected to reverse transcription using a PrimeScript RT Reagent kit with gDNA Eraser (Takara, Japan).

Candidate genes in the target region were predicted using the Williams 82 soybean reference genome GlymaWm82.a2.v1 (http://soybase.org). qRT-PCR was conducted to obtain the expression profiles of candidate genes using primers designed with Primer Premier 5.0. The housekeeping gene *Actin* was used as a control. The specific primers for each gene are listed in additional file Table S3.

qRT-PCR was performed with a CFX96 Real-Time PCR Detection System (Bio-Rad, USA) using a KAPA SYBR® FAST qPCR Kit (Kapa Biosystems). All reactions were carried out in 20-μl volumes containing 1 μl cDNA as a template. The thermal cycling conditions were as follows: 95 °C for 3 min, followed by 40 cycles of 95 °C for 10 s, 55.0–63.3 °C (depending on the gene) for 10 s and 72 °C for 30 s. Three independent biological repeats were performed to ensure accurate statistical analysis. The qRT-PCR data were evaluated using the 2^−ΔΔCt^ method [72].

## Supplementary information

**Additional file 1: Table S1.** Gene ontology (GO) enrichment analysis of the candidate genes. **Table S2.** The biological process and molecular function ontology of the candidate genes. **Table S3.** Primers of the candidate genes for real-time PCR.

**Additional file 2: Figure S1.** Twenty linkage groups of the soybean high-density genetic map. A high-density bin linkage map was constructed, covering 3032 cM, with an average distance of 0.81 cM between adjacent bins. The bin markers and their locations are shown on the right and left sides, respectively.

**Additional file 3: Figure S2.** Gene ontology (GO) enrichment analysis of the candidate genes of the *RpsGZ* locus. AgriGO (http://bioinfo.cau.edu.cn/agriGO/) was used to analyse the candidate genes of the *RpsGZ* locus, and significantly enriched GO categories under molecular functions are shown in orange and yellow boxes.

## Data Availability

The datasets supporting the results of this study are included in the manuscript. Soybean seeds are available from the Guangdong Subcenter of the National Center for Soybean Improvement, PR China.

## Abbreviations

bp: Base pair
CIM: Composite interval mapping
cM: Centimorgan
cv.: Cultivar
GO: Gene ontology
MAS: Molecular-assisted selection
NGS: Next-generation sequencing
PPR: *Phytophthora* root rot
*P. sojae*: *Phytophthora sojae*
RIL: Recombinant inbred line
SNP: Single nucleotide polymorphism
LOD: Log-likelihood
