# Supplementary material for: Fine mapping of a Phytophthora-resistance locus RpsGZ in soybean using genotyping-by-sequencing
Source: BMC Genomics. 2020 Apr 3;21:280. doi: 10.1186/s12864-020-6668-z (PMC7126358; doi:10.1186/s12864-020-6668-z)
Supplement: Supplementary file 1 — Additional file 1: Table S1. Gene ontology (GO) enrichment analysis of the candidate genes. Table S2. The biological process and molecular function ontology of the candidate genes. Table S3. Primers of the candidate genes for real-time PCR. [file 12864_2020_6668_MOESM1_ESM.pdf]

Table S1 Gene ontology (GO) enrichment analysis of the candidate genes

Ontology	GO ID	GO Term	Genes
Molecular function	GO:0009987	cellular process	*Glyma.03G036200,Glyma.03G035100,Glyma.03G035300 ,Glyma.03G036000*
	GO:0030554	adenyl nucleotide binding	*Glyma.03G034900, Glyma.03G034800, Glyma.03G035300, Glyma.03G034400, Glyma.03G036000, Glyma.03G034500*
	GO:0032553	ribonucleotide binding	*Glyma.03G034900, Glyma.03G034800, Glyma.03G035300, Glyma.03G034400, Glyma.03G036000*
	GO:0032559	adenyl ribonucleotide binding	*Glyma.03G034900, Glyma.03G034800, Glyma.03G035300, Glyma.03G034400, Glyma.03G036000, Glyma.03G034500*
	GO:0032555	purine ribonucleotide binding	*Glyma.03G034900, Glyma.03G034800, Glyma.03G035300, Glyma.03G034400, Glyma.03G036000, Glyma.03G034500*
	GO:0001883	purine nucleoside binding	*Glyma.03G034900, Glyma.03G034800, Glyma.03G035300, Glyma.03G034400, Glyma.03G036000, Glyma.03G034500*
	GO:0001882	nucleoside binding	*Glyma.03G034900, Glyma.03G034800, Glyma.03G035300, Glyma.03G034400, Glyma.03G036000, Glyma.03G034500*
	GO:0017076	purine nucleotide binding	*Glyma.03G034900, Glyma.03G034800, Glyma.03G035300, Glyma.03G034400, Glyma.03G036000*
	GO:0000166	nucleotide binding	*Glyma.03G034900, Glyma.03G034800, Glyma.03G035300, Glyma.03G034400, Glyma.03G036000, Glyma.03G034500*
	GO:0005488	binding	*Glyma.03G034800, Glyma.03G034900, Glyma.03G035300, Glyma.03G034400, Glyma.03G035200, Glyma.03G036000, Glyma.03G034500*

Table S2 The biological process and molecular function ontology of the candidate genes

Genes	GO terms
Glyma.03G034400	GO:0043531 ADP binding
Glyma.03G034500	GO:0043531 ADP binding
Glyma.03G034800	GO:0005515 protein binding
	GO:0043531 ADP binding
Glyma.03G034900	GO:0005515 protein binding
	GO:0043531 ADP binding
Glyma.03G035100	GO:0003678 DNA helicase activity
	GO:0000723 telomere maintenance
	GO:0006281 DNA repair
Glyma.03G035200	GO:0008270 zinc ion binding
	GO:0003677 DNA binding
Glyma.03G035300	GO:0006468 protein amino acid phosphorylation
	GO:0005524 ATP binding
	GO:0004672 protein kinase activity
Glyma.03G036000	GO:0006468 protein amino acid phosphorylation
	GO:0005524 ATP binding
	GO:0004672 protein kinase activity
Glyma.03G036200	GO:0015297 antiporter activity
	GO:0016020 membrane
	GO:0006855 multidrug transport
	GO:0015238 drug transmembrane transporter activity

Table S3 Primers of the candidate genes for real-time PCR

Gene ID	Upstream primers	ownstream primers	Tm(℃)
Glyma.03G034400	TGGCATCAAGTCCTTCCCTAA	GTGAGATGGAGAAGCCCTGTG	58
Glyma.03G034500	TGTCATTTCCAGGTGGTCGTT	AAGCAGGATCTCCTTCTGGTCTC	59
Glyma.03G034800	GATCTAGCCACATCACTCGG	ATGATACATCGTGCCTCCTC	54
Glyma.03G034900	ATTAAGGGATTGCTCGTCAG	CTCGGAGATTTGGAAAGGTA	54
Glyma.03G035300	TTTCAGCAGCCAACATACAGATT	CTGACAGGTTTCCAACAGCACAT	58
Glyma.03G035900	CAGATTACAGTCGGACGTAGGC	GATGAAGGATTTTCGGAAGAGA	56
Glyma.03G036000	AAGATCCTAACATGAGACCAAGC	AGAAAACAAGTGCCGAATAAACG	56
Actin	CGGTGGTTCTATCTTGGCATC	GTCTTTCGCTTCAATAACCCTA	55
