# Supplementary figures and images for: Fine mapping of a Phytophthora-resistance locus RpsGZ in soybean using genotyping-by-sequencing
Source: BMC Genomics. 2020 Apr 3;21:280. doi: 10.1186/s12864-020-6668-z (PMC7126358; doi:10.1186/s12864-020-6668-z)

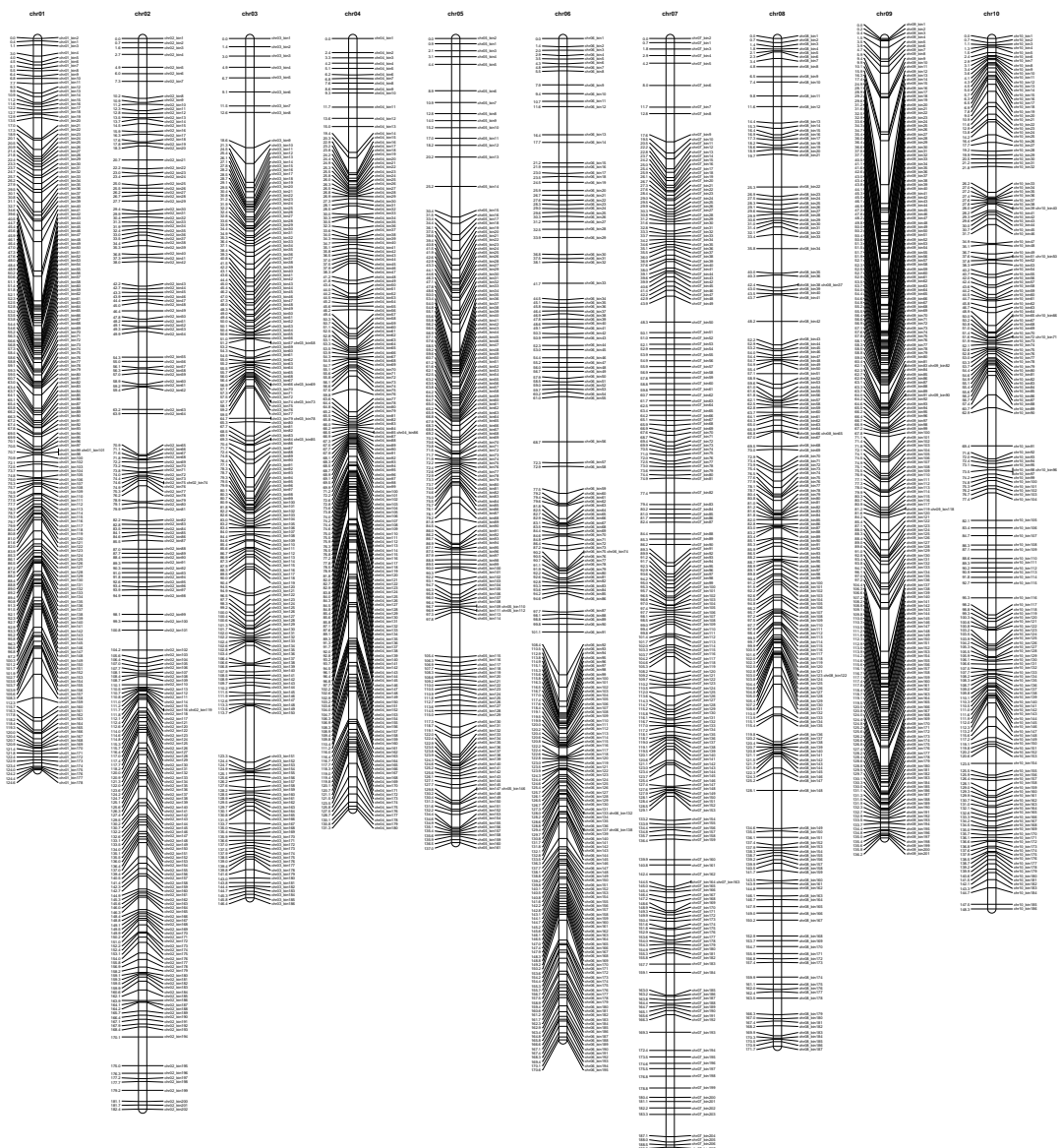

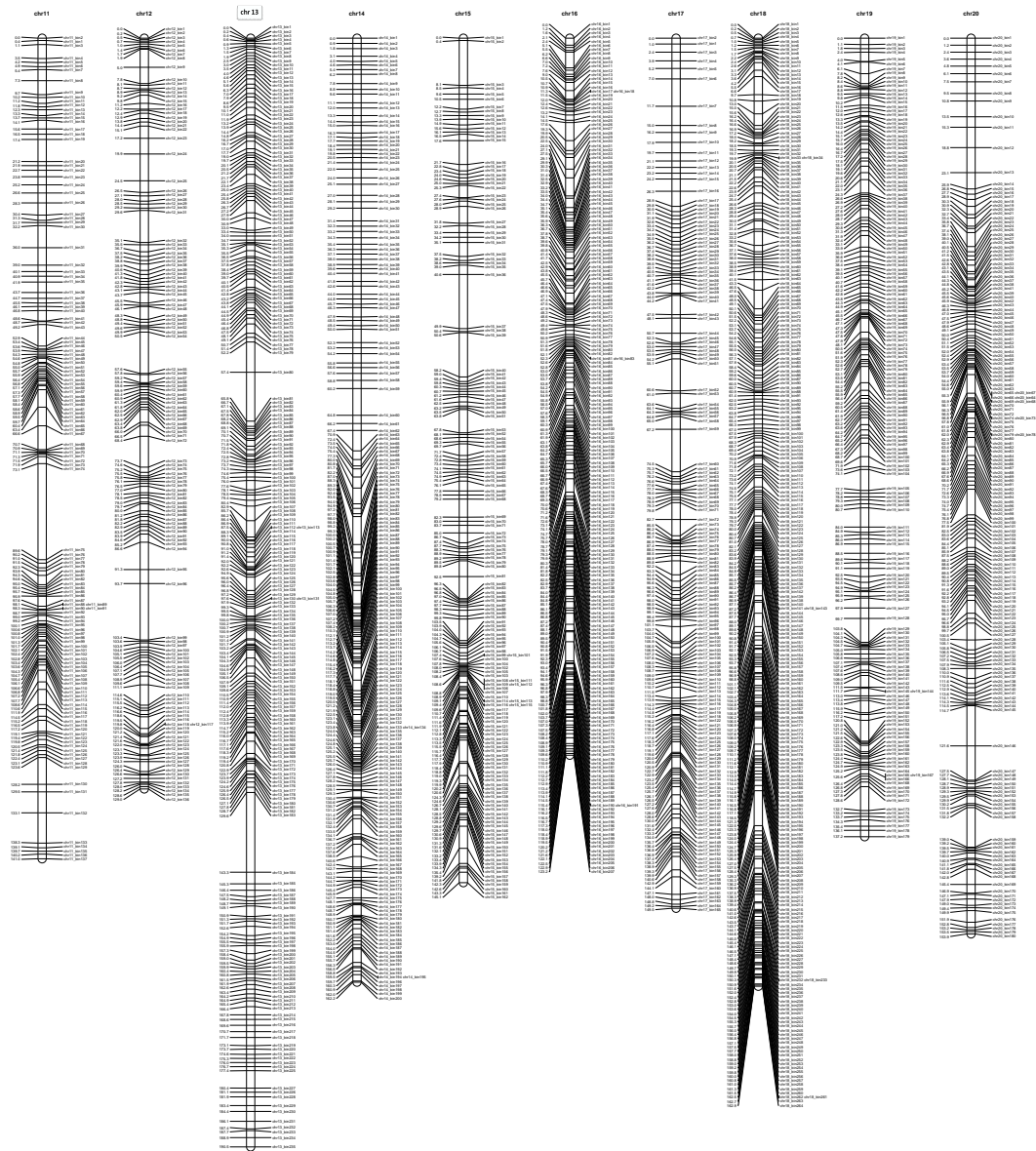

Supplement: Supplementary file 2 — Additional file 2: Figure S1. Twenty linkage groups of the soybean high-density genetic map. A high-density bin linkage map was constructed, covering 3032 cM, with an average distance of 0.81 cM between adjacent bins. The bin markers and their locations are shown on the right and left sides, respectively. [file 12864_2020_6668_MOESM2_ESM.pdf]

# Significance levels and Arrow types Diagram

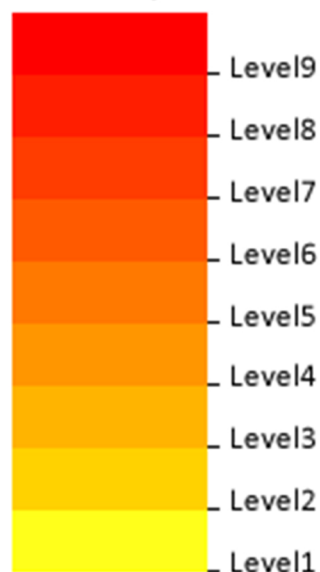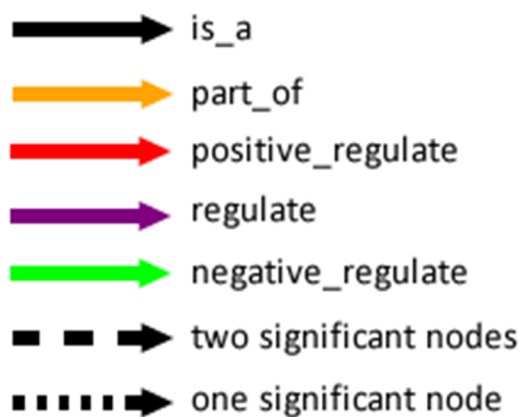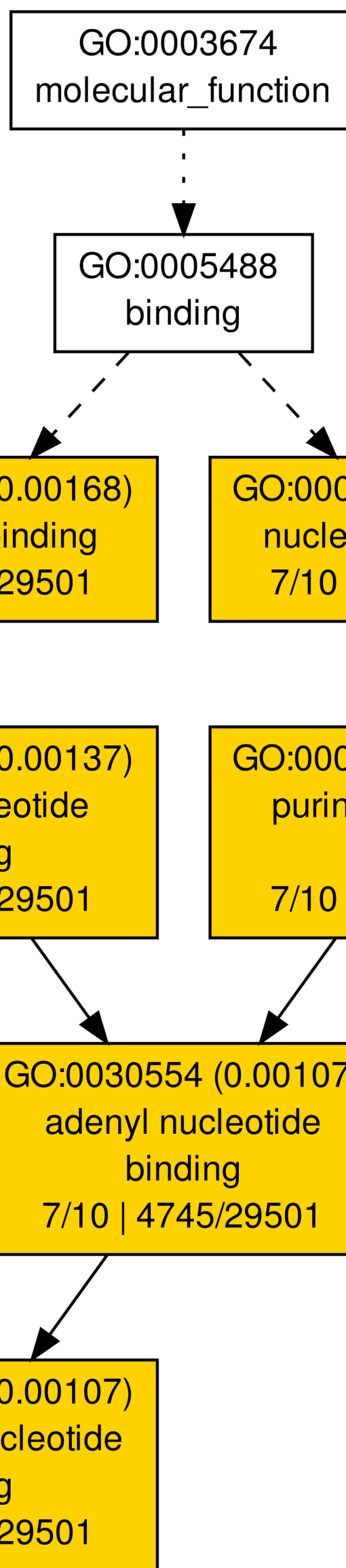

Supplement: Supplementary file 3 — Additional file 3: Figure S2. Gene ontology (GO) enrichment analysis of the candidate genes of the RpsGZ locus. AgriGO (http://bioinfo.cau.edu.cn/agriGO/) was used to analyse the candidate genes of the RpsGZ locus, and significantly enriched GO categories under molecular functions are shown in orange and yellow boxes. [file 12864_2020_6668_MOESM3_ESM.pdf]
